# Role of central kisspeptin and RFRP‐3 in energy metabolism in the male Wistar rat

**DOI:** 10.1111/jne.12973

**Published:** 2021-05-07

**Authors:** Fernando Cázarez‐Márquez, Jitske Eliveld, Wayne I. G. R. Ritsema, Ewout Foppen, Yvonne Bossenbroek, Simone Pelizzari, Valérie Simonneaux, Andries Kalsbeek

**Affiliations:** ^1^ Institute of Cellular and Integrative Neurosciences (INCI) Strasbourg France; ^2^ Netherlands Institute for Neuroscience (NIN) Amsterdam The Netherlands; ^3^ Laboratory of Endocrinology Amsterdam UMC Amsterdam Gastroenterology & Metabolism University of Amsterdam Amsterdam The Netherlands; ^4^ Department of Endocrinology and Metabolism Amsterdam UMC University of Amsterdam Amsterdam The Netherlands

**Keywords:** food intake, glucose homeostasis, hypothalamus, luteinising hormone, reproduction, RF‐amides

## Abstract

Kisspeptin (Kp) and (Arg)(Phe) related peptide 3 (RFRP‐3) are two RF‐amides acting in the hypothalamus to control reproduction. In the past 10 years, it has become clear that, apart from their role in reproductive physiology, both neuropeptides are also involved in the control of food intake, as well as glucose and energy metabolism. To investigate further the neural mechanisms responsible for these metabolic actions, we assessed the effect of acute i.c.v. administration of Kp or RFRP‐3 in ad lib. fed male Wistar rats on feeding behaviour, glucose and energy metabolism, circulating hormones (luteinising hormone, testosterone, insulin and corticosterone) and hypothalamic neuronal activity. Kp increased plasma testosterone levels, had an anorexigenic effect and increased lipid catabolism, as attested by a decreased respiratory exchange ratio (RER). RFRP‐3 also increased plasma testosterone levels but did not modify food intake or energy metabolism. Both RF‐amides increased endogenous glucose production, yet with no change in plasma glucose levels, suggesting that these peptides provoke not only a release of hepatic glucose, but also a change in glucose utilisation. Finally, plasma insulin and corticosterone levels did not change after the RF‐amide treatment. The Kp effects were associated with an increased c‐Fos expression in the median preoptic area and a reduction in pro‐opiomelanocortin immunostaining in the arcuate nucleus. No effects on neuronal activation were found for RFRP‐3. Our results provide further evidence that Kp is not only a very potent hypothalamic activator of reproduction, but also part of the hypothalamic circuit controlling energy metabolism.

## Graphical abstract

In the past 10 years, it has become clear that apart from their role in reproductive physiology the RF‐amides Kisspeptin (Kp) and (Arg)(Phe) related peptide 3 (RFRP‐3) are also involved in the control of food intake, as well as glucose and energy metabolism. To investigate further the neural mechanisms responsible for these metabolic actions, we assessed the effect of acute i.c.v. administration of Kp or RFRP‐3 in ad lib. fed male Wistar rats on feeding behaviour, glucose and energy metabolism, circulating hormones (luteinising hormone, testosterone, insulin and corticosterone) and hypothalamic neuronal activity. Our results provide further evidence that Kp is not only a very potent hypothalamic activator of reproduction, but also part of the hypothalamic circuit controlling energy metabolism.

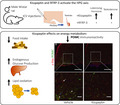

## 1 INTRODUCTION

The (Arg)(Phe)‐amide peptides, kisspeptin (Kp) and (Arg)(Phe) related peptide 3 (RFRP‐3), are two hypothalamic peptides that are well known for modulating reproductive activity in mammals. Kp has been described as a potent activator of GnRH neuronal activity, leading to increased secretion of gonadotrophins and sexual hormones in all mammalian species investigated, including humans.^1^, ^2^ By contrast, the role of RFRP‐3 is still under debate because stimulatory, inhibitory or absent effects have been reported according to species, sex and seasons.^3^, ^4^

Reproduction is a very expensive process in terms of energetic needs, which makes it essential for mammals to match the timing of reproduction with an optimal energetic and metabolic status. Thus, it is not that surprising that, recently, Kp and RFRP‐3 have also been linked to the control of food intake, body weight regulation and glucose homeostasis.^5^, ^6^, ^7^ The scarce and scattered data so far point towards RFRP‐3 having an orexigenic effect in different mammalian species^7^, ^8^, ^9^, ^10^, ^11^ and Kp having an anorexigenic effect.^10^, ^12^ Regarding glucose homeostasis, it has been shown that female mice with a knockout (KO) for the Kp receptor Kiss1r are glucose intolerant,^5^ whereas i.p. administration of RFRP‐3 changed circulating glucose concentrations and insulin receptor and glucose transporter expression in testis and adipose tissue.^13^ Interestingly, it has been found that one in three men with type 2 diabetes present detrimental effects on gonadal activity (hypogonadism)^14^ and testosterone replacement has positive effects on metabolic syndrome survival rates.^15^, ^16^

Within the hypothalamus, the arcuate nucleus (ARC), a brain region well known to receive and integrate many metabolic signals from the periphery, shows a high expression of both Kp^17^ and RFRP‐3^18^, ^19^ receptors. The two main populations of neurones within the ARC that are responsible for the control of energy metabolism and glucose homeostasis are the orexigenic neuropeptide Y (NPY)/agouti‐related peptide (AGRP)‐expressing neurones and the anorexigenic pro‐opiomelanocortin (POMC)/cocaine‐ and amphetamine‐regulated transcript (CART)‐expressing neurones.^20^ In our previous studies in the seasonal Djungarian hamster we showed that central administration of Kp increased body weight as well as NPY‐ and POMC expression, whereas RFRP‐3 increased food intake, body weight and circulating levels of leptin and insulin, without changing NPY‐ and POMC expression in the ARC.^7^, ^11^ In the present study, we tested the hypothesis that Kp and RFRP‐3 would also affect energy metabolism in male Wistar rats. Therefore, we assessed the central effects of Kp and RFRP‐3 on feeding behaviour, energy metabolism and glucose homeostasis in this species and revealed possible hypothalamic pathways involved in the reported metabolic effects.

## 2 MATERIALS AND METHODS

### 2.1 Animals

Adult male Wistar rats (Charles River, Sulzfeld, Germany) weighing 250‐280 g at the start of the experiment were used in all experiments. Animals were housed in individual cages in an enriched environment with a wooden stick under a 12:12 hour light/dark photocycle (lights on 7.00 am; =ZT0). Food (24% protein, 58% carbohydrate and 18% fat) (Teklad global diet 2918; Envigo, Indianapolis, IN, USA) and water were provided ad lib. After arrival, rats could adapt to the animal facility with constant temperature (21 ± 2°C) and humidity (50 ± 5%) for at least 1 week. All experimental procedures performed were approved by the Animal Ethics Committee of the Royal Dutch Academy of Arts and Sciences (KNAW, Amsterdam, The Netherlands) and were in accordance with the guidelines on animal experimentation of the Netherlands Institute for Neuroscience (NIN).

### 2.2 Surgery

To infuse either RFRP‐3 or Kp in the i.c.v. space of the central nervous system, a unilateral brain cannula (Plastic One , Dusseldorf, Germany) reaching the lateral ventricle was implanted. Surgery was conducted under anaesthesia consisting of an i.m. injection of a mix of ketamine (80 mg kg^‐1^; Eurovet Animal Health, Bladel, The Netherlands) and xylazine (8 mg kg^‐1^; Bayer Health Care, Mijdrecht, The Netherlands). The coordinates were defined using the rat brain atlas^21^ as a reference: −0.8 mm anteroposterior, +2.0 mm lateral from bregma and −3.2 mm ventral from the dura. In some of the animals, silicon catheters were surgically implanted into the right jugular vein and the left carotid artery for i.v. infusion and blood sampling, respectively.^22^ Brain cannula and catheters were fixed to the skull using dental cement. A cannula dummy was used to seal the guide cannula maintaining it open until the infusion. A metallic connector that could be attached to a chain swivel was added to the dental cement, which allowed us to execute the experiment without handling of the animals during the experiment. The animals received carprofen as a postoperative analgesic (2.5 mg kg^‐1^; Zoetis, Parsippany‐Troy Hills, NJ, USA). The rats were allowed to recover for at least 10 days after the surgery, with experiments only being started after they had reached their initial pre‐operative body weight again.

#### 2.2.1 Experimental set‐up for indirect calorimetry

Seven days after surgery, animals were single housed in metabolic cages (TSE, Bad Homburg, Germany) for three consecutive days. Day 1 was aimed for habituation, day 2 for a baseline measurement and then on the morning of day 3 animals were i.c.v. injected (~ZT5) and the automatised measurements continued for 24 hours. Animals had ad lib. access to water and food from hanging bottles and baskets, respectively. Food and water intake, respiratory exchange ratio (RER), energy expenditure and locomotor activity were recorded continuously during these 3 days. On the afternoon of day 4, animals were moved back to their regular housing conditions.

### 2.3 Intracerebroventricular peptide infusion

Every animal received a cross‐treatment with vehicle (sterile NaCl 0.9%) and Kp (3 nmol/5 µL; Rat Kp10 sequence; ToCris Bioscience, Bristol, UK; n = 15) or RFRP‐3 (50 or 250 pmol/5 µL; Rat RFRP‐3 sequence; Caslo Laboratory, Lyngby, Denmark; 50 pmol, n = 8; 250 pmol, n = 7). The initial dose of 50 pmol RFRP‐3 was based on the 100 ng dose of Johnson et al^8^; when we found no effects with 50 pmol, we increased the dose to 250 pmol. Brain injections were performed at a rate of 1 µL min^‐1^ and patency was corroborated by tracking the movement of a small air bubble. All animals were injected between ZT4.5 and ZT5.5. Animals were handled for 3‐5 min day^‐1^ for at least 4 days before each i.c.v. injection to habituate them to the procedure. Animals were allowed to recover for 7 days after each i.c.v. injection.

### 2.4 Perfusion and peripheral tissue sampling

At the end of the experiment, rats were given a third i.c.v. injection with either vehicle or one of the RF‐amides and killed 1 hour after under an overdose of i.p. injected pentobarbital. A blood sample was taken by heart puncture and then animals were perfused intracardially with 150 mL of NaCl 0.9%. Next, animals were perfused with 100 mL of formaldehyde 4%, and then brains were removed and post‐fixed overnight. Brains were then transferred to 30% sucrose in Tris‐buffered saline for cryoprotection. Brains were sliced at 35 µm thickness with a Cryostar NX50 cryostat (Thermo Fisher Scientific, Waltham, MA, USA) and stored in cryoprotectant (30% ethylene glycol, 30% glycerol, 40% phosphate‐buffered saline) for later immunostainings.

#### 2.4.1 Experimental set‐up for measuring endogenous glucose production (EGP)

**FIGURE 1:**
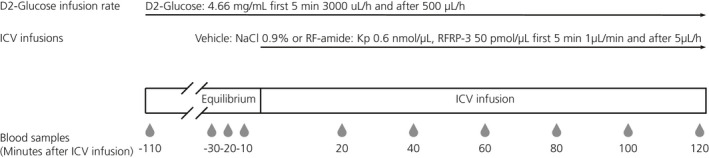
Experimental protocol of endogenous glucose production during RF‐amides or vehicle i.c.v. treatment. The droplets and numbering in the bottom row indicate the timing of blood sampling in minutes

We used [6,6‐^2^H_2_]glucose (D2‐glucose) to evaluate EGP during a 2‐hour continuous i.c.v. infusion of either Kp or RFRP‐3. Experiments were performed using a cross‐over design with at least 1 week of recovery in between. On the evening prior to the EGP evaluation, animals were attached to a counterbalanced swivel that allowed blood sampling without handling the animal. On the following morning, food was removed at ZT0 and the arterial and venous catheters were connected to a tubing line filled with heparinised (1%) saline (Figure 1). At ZT3, a blood sample was taken for basal measurements and the tubing for the i.c.v. infusion was filled up, connected to the cannula injector and sealed to avoid leaking into the ventricular space prior to the start of the brain infusion. At ZT4, the D2 glucose infusion was started using a primed‐continuous administration protocol, starting first with a 5‐minute infusion at a rate of 3000 µL h^‐1^ and then continued with a rate of 500 µL h^‐1^ until the end of the experiment. Ninety minutes later at ZT5.5, when a steady‐state was reached, 200‐µL blood samples were taken every 10 minutes for a total of three samples to calculate the basal level of EGP before the start of the brain infusion. Finally, at ZT6, a primed i.c.v. infusion was started. Either Kp (0.6 nmol µL^‐1^), RFRP‐3 (50 pmol µL^‐1^) or vehicle (NaCl 0.9%) were infused at a rate of 1 µL min^‐1^ for the first 5 minutes, which was then decreased to 5 µL h^‐1^ for the remainder of the experiment. Blood samples were taken every 20 minutes over 2 hours to calculate the change in EGP during the brain infusion. After 2 hours, the i.c.v. and D2‐glucose infusions and blood sampling were stopped and all external tubing was removed. Animals were immediately returned to ad lib. water and food access. At the end of the experiment, animals were killed, then fresh brains were collected, frozen and sliced with a cryostat to verify the cannula placement.

### 2.5 Plasma hormonal measurements

Blood samples were centrifuged at 1700 g for 15 minutes at 4°C and plasma was collected for hormone and labelled glucose measurements. Glucose concentrations were measured directly from blood samples with a glucometer (Abbott Laboratories, Chicago, IL, USA). Plasma [6,6‐^2^H_2_] glucose enrichment was measured by gas chromatography‐mass spectrometry and EGP was calculated using the methods of Steele.^23^ Corticosterone (MP Biomedicals, Santa Ana, CA, USA) and insulin (Millipore, Burlington, MA, USA) levels were determined by radioimmunoassays in accordance with the manufacturer's instructions, the luteinising hormone (LH) level was determined using an enzyme‐linked immunosorbent assay^24^ and testosterone levels were measured using isotope dilution‐liquid chromatography‐tandem mass spectrometry.^25^

### 2.6 Immunostaining

Brain slices containing hypothalamic nuclei were selected using bregma as reference according to the rat brain atlas from Paxinos and Watson (Table 1).^21^ Cellular activity was evaluated by c‐Fos immunostaining. Sections were incubated with rabbit c‐Fos antibody (dilution 1:1000; sc‐52; Santa Cruz Biotechnology, Santa Cruz, CA, USA) for 1 hour at room temperature followed by overnight at 4°C, then sections were incubated with a goat biotinylated antibody anti‐rabbit (diltion 1:500; BA1000; Vector Labs, Burlingame, CA, USA) for 90 minutes. The signal was made visible by incubation with the avidin‐biotin complex (dilution 1:500; Vector Labs) for 1 hour and revealed with 3,3′‐diaminobenzidine (0.5 mg mL^‐1^), H_2_O_2_ (0.03%) and nickel ammonium sulfate (230 μg mL^‐1^). Finally, sections were mounted on slides, dehydrated in increasing concentrations of ethanol and xylene, and cover slipped with Entellan (Millipore).

**TABLE 1:** Brain nuclei stained for c‐Fos

Distance from bregma (mm)	Abbreviation	Nucleus or nuclei
0.48	OVLT	Organum vasculosum of the laminae terminalis
0.24	aMnPO	Anterior part of the median preoptic nucleus (MnPO)
0.12	AVPV	Anteroventral periventricular nucleus
0	MPA	Medial preoptic area
−0.12	pMnPO	Posterior part of the MnPO
−0.48	aSCN	Anterior part of the suprachiasmatic nucleus (SCN)
−0.72	mSCN	Medial part of the SCN
−0.96	pSCN	Posterior part of the SCN
−1.8	aARC	Anterior part of arcuate nucleus (ARC)
−2.64	mARC	Medial part of the ARC
−3.84	pARC	Posterior part of the ARC

Double stainings for c‐Fos and POMC were performed by staining one section at the anterior, medial and posterior part of the ARC (aARC, mARC and pARC) per animal. Sections were incubated with primary antibodies, rabbit anti‐POMC (dilution 1:4000; H‐029‐30; Phoenix Pharmaceuticals Inc., Belmont, CA, USA) and sheep anti c‐Fos (dilution 1:1000; PA1‐18329; Thermo Fisher Scientific) for 1 hour at room temperature and overnight at 4°C. Then, sections were incubated with a donkey anti‐sheep (Alexa 594; A‐11016; Invitrogen, Carlsbad, CA, USA) and a donkey anti‐rabbit (Alexa 488; A32723; Invitrogen) coupled to fluorophore. Sections were mounted on slides and cover slipped with mounting media containing 4ʹ,6‐diamidino‐2‐phenylindole (DAPI) (H‐1200; Vectashield; Vector Labs).

For all experiments, the specificity of the first antibody was assessed by verifying that removal of the primary antibody resulted in an absence of immunostaining. In addition, the specificity of the anti‐POMC was verified by pre‐absorption controls on ARC brain sections containing POMC neurones, where staining was abolished.^26^, ^27^ The specificity of the Santa Cruz rabbit c‐Fos antibody (Sc‐52) was demonstrated by Magno et al^28^ and that for the Thermo Fisher sheep c‐Fos antibody (PA1‐18329) was demonstrated by Wang et al.^29^

### 2.7 Image analysis

Sections stained for c‐Fos immunostaining were photographed with a CCD camera (Model 77CE; Sony, Tokyo, Japan) attached to a microscope (Axioskop with Plan‐NEOFLUAR objectives; Carl Zeiss GmbH, Carl Zeiss, Oberkochen, Germany) using a 10× 0.63 objective. Double immunofluorescence was visualised with a confocal microscope (TCS SP8 SMD; Leica, Wetzlar, Germany) using a 40x objective. For each staining analysed, images of the areas of interest were taken at the same time under identical lighting or laser set‐ups for all animals. A person unaware of the treatments performed the quantification of either c‐Fos expression, POMC staining and co‐localisation using the ImageJ software (NIH, Bethesda, MD, USA). Positive c‐Fos staining was evaluated by setting a threshold to a mean size of the nuclear positive staining. Then the mean size was used to threshold the quantification for the rest of the images. A rectangle or circle of the size of the brain area of interest was drawn and superimposed accordingly. The POMC‐immunoreactive area was evaluated by using a set threshold and quantifying the same sampled area size for every level of the ARC. Double immunostaining was evaluated by counting exclusively POMC positive cells that contained both, c‐Fos and DAPI in the nucleus.

### 2.8 Statistical analysis

Only data from animals with correct cannula placements and injections corroborated for patency were included in the data analysis. All data showed normal distribution and homogeneous variance according the Shapiro‐Wilk and Levene's tests and are represented as the mean ± SEM. Graphs and statistical analyses were conducted with Prism, version 8 (GraphPad Software Inc., San Diego, CA, USA). EGP measurements were analysed by repeated measures two‐way ANOVA and Bonferroni’s or Tukey’s post‐hoc honestly significant difference test. Hormonal levels and neuro‐anatomical results were compared by Student's *t*‐test. TSE data (food and water consumption, RER, energy expenditure and locomotor activity) were analysed by two‐way repeated measures ANOVA and Bonferroni’s post‐hoc honestly significant difference test. Delta values were calculated as the difference compared to the mean value of the three pre‐infusion samples. *P* < 0.05 was considered statistically significant.

## 3 RESULTS

To investigate the effects of Kp and RFRP‐3 on glucose and energy metabolism, two different experimental techniques and two slightly different experimental set‐ups were used. In Experiment 1, we used indirect calorimetry in so‐called metabolic cages (TSE) to measure the effects of Kp and RFRP‐3 on food and water intake, locomotor activity, energy expenditure and the RER. In Experiment 2, we used a chronic i.v. infusion with D2‐glucose to evaluate the effects on EGP. In Experiment 1, both RF‐amides were administered i.c.v. as a bolus injection, whereas, in Experiment 2, they were administered during a 2‐hour continuous i.c.v. infusion.

### 3.1 Experiment 1: Indirect calorimetry

Only the animals that successfully received both i.c.v. injections (vehicle and RF‐amide) were included in the analysis of food and water intake, RER, energy expenditure and locomotor activity; 9/15 for Kp and 9/15 for RFRP‐3 (n = 5 for 50 pmol, n = 4 for 250 pmol). During the third i.c.v. injection, aimed for tissue sampling, one animal from the RFRP group had to be excluded as a result of a failure of the i.c.v. injection.

#### 3.1.1 RF‐amide effects on the hypothalamic‐pituitary‐gonadal (HPG) axis, insulin and corticosterone

**FIGURE 2:**
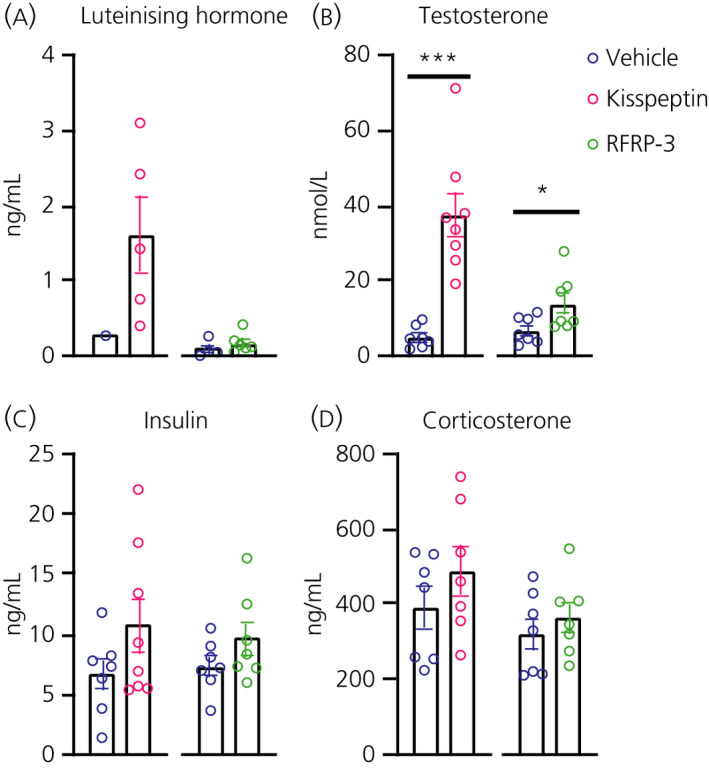
Effect of central kisspeptin (Kp) and RFRP‐3 on circulating hormones in male Wistar rats. Luteinising hormone (A), testosterone (B), insulin (C) and corticosterone (D) plasma levels were measured 1 hour after the i.c.v. injection of vehicle (NaCl 0.9%, blue circles), 3 nmol Kp (pink circles) or 250 pmol RFRP‐ = 7 or 8 animals per experimental group, with the scattered dots representing individual data values. For luteinising hormone, 13 out of 29 samples fell in the non‐detectable range, as also reported previously for male rodents. ***P < 0.001, *P < 0.05 after Student's *t*‐test when comparing the i.c.v. peptide infusion vs its respective i.c.v. vehicle control infusion

First, we verified the effect of both RF‐amides on the activity of the HPG axis. One hour after i.c.v. injection, both Kp and RFRP‐3 increased systemic testosterone levels compared to vehicle treated rats, although with a less potent effect of RFRP‐3 as compared to that of Kp (Kp: *P* = 0.001, RFRP‐3: *P* = 0.045) (Figure 2B). Plasma LH, insulin and corticosterone levels did not show statistically significant effects after the peptide treatment (Figure 2A,C,D).

#### 3.1.2 Central Kp injection decreases food intake

**FIGURE 3:**
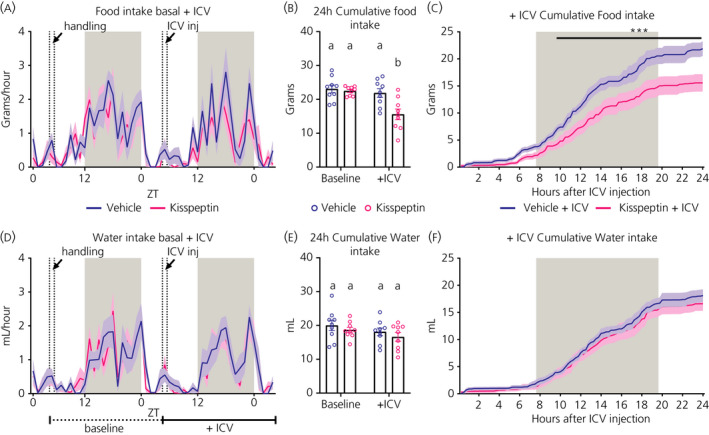
Effect of i.c.v. injection of kisspeptin (Kp) (3 nmol) or vehicle (NaCl 0.9%) on food and water intake in male Wistar rats. Hourly food (A) and water (D) intakes are plotted for the baseline and experimental (+i.c.v.) days, with data presented as the mean (solid line) ± SEM (shaded area) of n = 9 animals. Total 24‐hour food (B) and water (E) intake were calculated for the baseline and experimental day, as indicated by the horizontal lines below (D). Different lowercase letters in (B,E) indicate that the groups are statistically different after two‐way ANOVA and Tukey's post‐hoc honestly significance test. Cumulative food (C) and water (F) intake during the experimental day with a 15‐minute resolution during 24 hours and starting immediately after the Kp or vehicle injection. ***P < 0.001 statistical difference between Kp and vehicle after two‐way ANOVA and Bonferroni's post‐hoc honestly significance. ZT0 = lights ON, ZT12 = lights OFF. Grey background indicates the dark period. Handling or i.c.v. injection occurred between the two vertical dashed lines

Rats injected with 3 nmol Kp exhibited a decrease in 24‐hour food intake compared to the previous baseline day, as well as compared to their vehicle treatment (Figure 3A‐C). Both the comparison “Kp i.c.v. vs vehicle i.c.v.” and “Kp i.c.v. vs Kp baseline” revealed a significant effect of Treatment, respectively *P* = 0.0006 and *P* = 0.0059 (Table 2). Water intake was not changed after Kp injection (Figure 3D‐F and Table 2). By contrast, neither the 50 pmol (Table S1; see also Supporting information, Figure S1), nor 250 pmol (Table S2; see also Supporting information, Figure S2) i.c.v. injections of RFRP‐3 significantly changed food or water intake. In addition, when the two RFRP‐3 experiments were combined (n = 9), no significant effects on food or water intake were found (see Supporting information, Figure S3 and Table S3).

**TABLE 2:** Effects of i.c.v. kisspeptin injection (3 nmol) on metabolic outcomes

Comparison	Parameter	Treatment	Time	Interaction
Baseline vs vehicle i.c.v.	Cumulative food intake	*F*_1,8_ = 0.9580 *P* = 0.356	***F*_95,760_ = 206.5** ***P* < 0.0001**	***F*_95,760_ = 1.337** ***P* = 0.023**
	Cumulative water intake	*F*_1,8_ = 2.306 *P* = 0.167	***F*_95,760_ = 132.6** ***P* < 0.0001**	***F*_95,760_ = 3.595** ***P* < 0.0001**
	Locomotor activity	*F*_1,8_ = 4.798 *P* = 0.060	***F*_95,760_ = 6.120** ***P* < 0.0001**	*F*_95,760_ = 1.257 *P* = 0.058
	RER	*F*_1,8_ = 1.189 *P* = 0.307	***F*_95,760_ = 13.00** ***P* < 0.0001**	***F*_95,760_ = 1.571** ***P* = 0.0008**
	Heat	*F*_1,8_ = 0.5588 *P* = 0.476	***F*_95,760_ = 6.942** ***P* < 0.0001**	*F*_95,760_ = 0.9260 *P* = 0.675
Baseline vs Kp10 i.c.v.	Cumulative food intake	***F*_1,8_** = **13.84** ***P* = 0.006**	***F*_95,760_ = 264.5** ***P* < 0.0001**	***F*_95,760_ = 9.056** ***P* < 0.0001**
	Cumulative water intake	*F*_1,8_ = 1.038 *P* = 0.338	***F*_95,760_ = 259.7** ***P* < 0.0001**	*F*_95,760_ = 0.8811 *P* =.779
	Locomotor activity	***F*_1,8_** = **13.04** ***P* = 0.007**	***F*_95,760_** = **6.759** ***P* < 0.0001**	***F*_95,760_ = 1.675** ***P* = 0.0001**
	RER	***F*_1,8_** = **13.48** ***P* = 0.006**	***F*_95,760_ = 12.87** ***P* < 0.0001**	***F*_95,760_ = 3.121** ***P* < 0.0001**
	Heat	*F*_1,8_ = 4.479 *P* =.067	***F*_95,760_ = 6.288** ***P* < 0.0001**	***F*_95,760_ = 1.779** ***P* < 0.0001**
Vehicle i.c.v. vs Kp10 i.c.v.	Cumulative food intake	***F*_1,8_** = **30.19** ***P* =.0006**	***F*_95,760_** = **117.9** ***P* < 0.0001**	***F*_95,760_ = 12.39** ***P* < 0.0001**
	Cumulative water intake	*F*_1,8_ = 1.105 *P* = 0.324	***F*_95,760_** = **139.8** ***P* < 0.0001**	*F*_95,760_ = 0.5516 *P* = 0.999
	Locomotor activity	*F*_1,8_ = 0.8578 *P* = 0.382	***F*_95,760_** = **6.409** ***P* < 0.0001**	*F*_95,760_ = 0.6796 *P* = 0.991
	RER	***F*_1,8_** = **25.42 *P* = 0.001**	***F*_95,760_** = **12.78** ***P* < 0.0001**	*F*_95,760_ = 1.169 *P* = 0.141
	Heat	*F*_1,8_ = 0.04709 *P* = 0.834	***F*_95,760_** = **5.061** ***P* < 0.0001**	*F*_95,760_ = 0.8237 *P* = 0.883

Abbreviations

Kp, kisspeptin; RER, respiratory exchange ratio.

Significant values are indicated in bold.

#### 3.1.3 Central Kp injection decreases the RER

**FIGURE 4:**
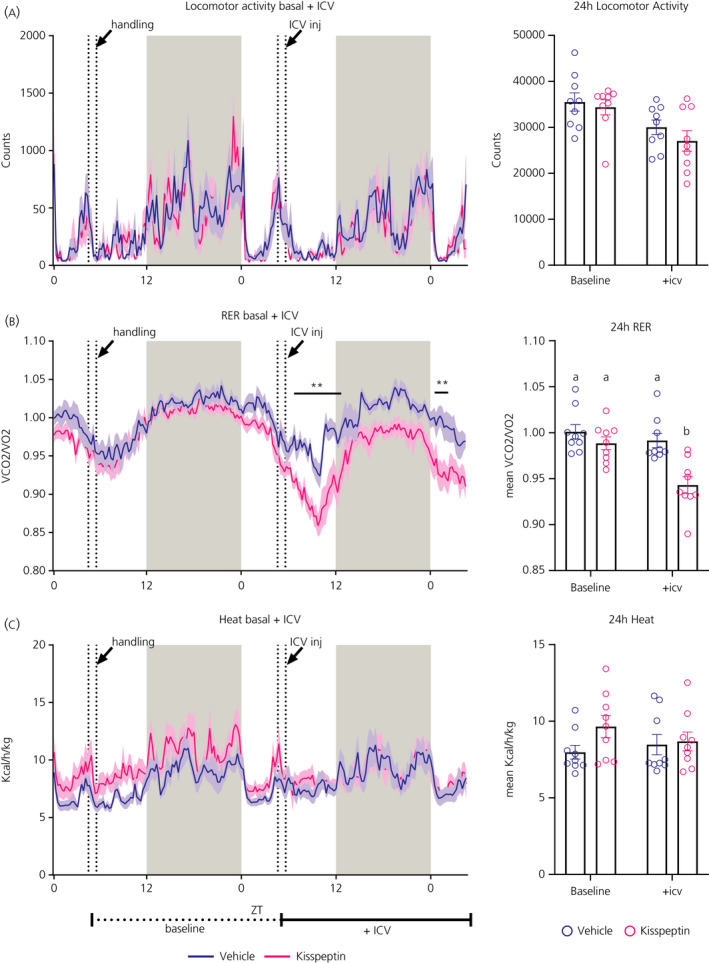
Effects of i.c.v. kisspeptin (Kp) (3 nmol) or vehicle (NaCl 0.9%) on locomotor activity, respiratory exchange ratio (RER) and energy expenditure in male Wistar rats. Locomotor activity (A), RER (B) and energy expenditure or heat (C) are plotted for the baseline and experimental (+i.c.v.) day, with data presented as the mean (solid line) ± SEM (shaded area) of n = 9 animals; **P < 0.01 statistical difference between Kp and vehicle after two‐way ANOVA and Bonferroni's post‐hoc honestly significance test. Total locomotor activity, mean RER and energy expenditure were calculated during 24 hours for the baseline and experimental day, as indicated by the horizontal lines below (C). Different lowercase letters indicate that the groups are statistically different after two‐way ANOVA and Tukey's post‐hoc honestly significance test. ZT0 = lights ON, ZT12 = lights OFF. Grey shaded area indicates dark phase. Handling or i.c.v. injection occurred between the two dashed lines

The RER value indicates the main fuel source utilised by the body for energy production. This ratio fluctuates over the daily cycle and is close to 1.0 during the dark (ie, feeding) phase when mainly carbohydrates are oxidised, but decreases during the light (ie, sleeping) period when animals are fasting and lipids become the preferential source of fuel. The i.c.v. injection of Kp significantly decreased the RER (Figure 4B). Repeated measures‐ANOVA showed significant Treatment effects for the “Kp i.c.v. vs vehicle i.c.v.” and “Kp i.c.v. vs Kp baseline” comparison, *P* = 0.0010 and *P* = 0.0063, respectively (Table 2). In addition, the 24‐hour mean RER level was decreased in the Kp–i.c.v. group (Figure 4B). By contrast, energy expenditure was not significantly affected by Kp (Figure 4C). Locomotor activity showed a significant Treatment effect in the “Kp i.c.v. vs Kp baseline“ comparison (*P* = 0.0069) (Figure 4A and Table 2), but no differences in 24‐hour total activity were detected (Figure 4A). None of the doses of RFRP‐3, either separately or combined, significantly changed RER, locomotor activity or energy expenditure (see Supporting information, Figures S4 and S5 and Tables S1‐S3).

### 3.2 Experiment 2: EGP

Ten animals from the Kp experiment (vehicle n = 4, Kp n = 6) and 11 animals from the RFRP‐3 experiment (vehicle n = 5, RFRP‐3 n = 6) completed the experiment with correct infusions and complete blood samplings. None of the plasma measurements showed a statistically significant difference when comparing the RF‐amide vs vehicle groups before the start of the i.c.v. infusion (Table 3).

**TABLE 3:** Basal levels of plasma measurement before vehicle or RF‐amide i.c.v. infusion (mean ± SEM)

Experiment		Vehicle i.c.v.	RF‐amide	*P* value
Kisspeptin i.c.v. infusion	Luteinising hormone (ng mL^‐1^)	−0.2479 ± 0.2593	0.03367 ± 0.08949	0.262
	Testosterone (nmol L^‐1^)	3.475 ± 1.040	7.383 ± 1.985	0.173
	Corticosterone (ng mL^‐1^)	65.60 ± 30.45	81.17 ± 40.82	0.863
	Insulin (ng mL^‐1^)	2.110 ± 0.3548	1.437 ± 0.1749	0.095
	Glucose (mmol L^‐1^)	3.883 ± 0.2327	3.933 ± 0.1805	0.868
	Endogenous glucose production (µmol kg·min^‐1^)	73.56 ± 9.345	63.49 ± 2.600	0.248
RFRP‐3 i.c.v. infusion	Luteinising hormone (ng mL^‐1^)	−0.1300 ± 0.08961	−0.1767 ± 0.1818	0.834
	Testosterone (nmol L^‐1^)	3.520 ± 0.5826	5.900 ± 2.452	0.411
	Corticosterone (ng mL^‐1^)	34.80 ± 14.08	24.67 ± 7.575	0.522
	Insulin (ng mL^‐1^)	1.288 ± 0.07186	1.475 ± 0.1245	0.250
	Glucose (mmol L^‐1^)	3.953 ± 0.04295	4.100 ± 0.08735	0.192
	Endogenous glucose production (µmol kg·min^‐1^)	61.09 ± 3.674	63.14 ± 7.244	0.8191

#### 3.2.1 Effects of RF‐amide infusion on LH, testosterone, insulin and corticosterone secretion

**FIGURE 5:**
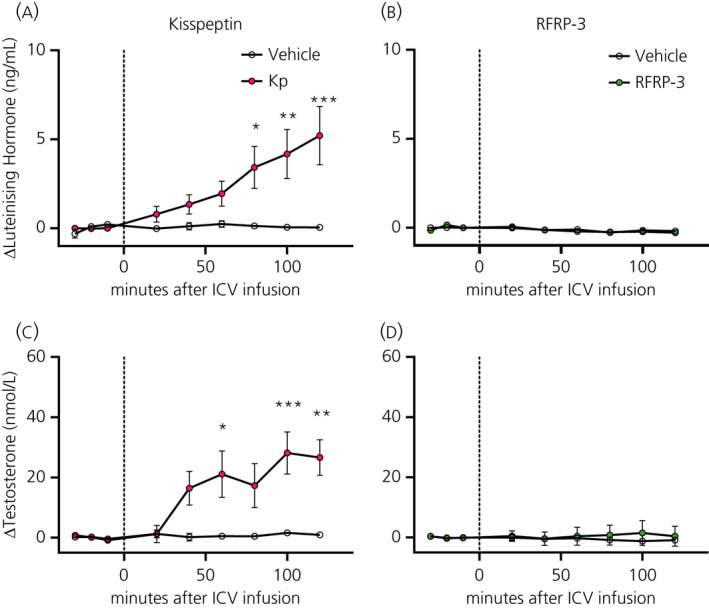
Effect of central kisspeptin (Kp) and RFRP‐3 on hypothalamic‐pituitary‐gonadal axis activity. Luteinising hormone (A,B) and testosterone (C,D) plasma levels were measured at −30, −20, −10, 20, 40, 60, 80, 100 and 120 minutes after the start of the i.c.v. infusion of Kp (3 nmol h^‐1^, pink circles), RFRP‐3 (50 pmol h^‐1^, green circles) or vehicle (NaCl 0.9%, open circles). Data are the mean ± SEM of n = 4‐6 for Kp and n = 5‐6 for RFRP‐3 treated animals per experimental group. *P < 0.05, **P < 0.01, ***P < 0.001 statistical difference between Kp and vehicle after two‐way RM ANOVA and Bonferroni's post‐hoc honestly significance test

**FIGURE 6:**
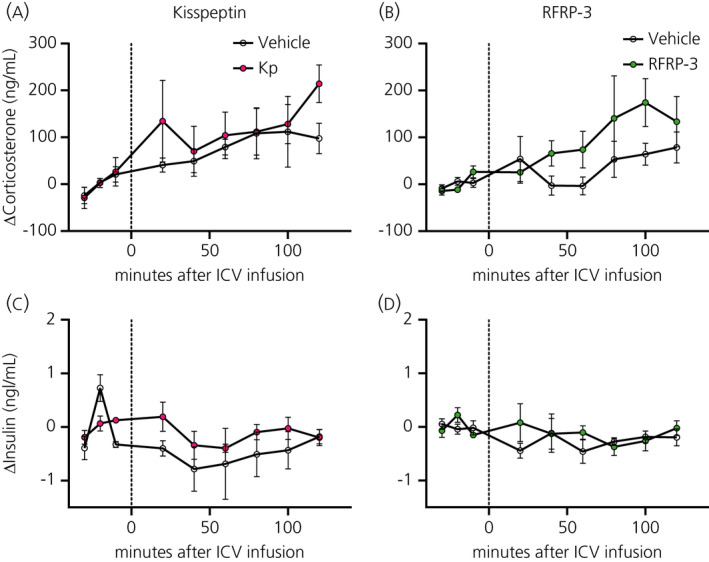
Effect of central kisspeptin (Kp) and RFRP‐3 on corticosterone and insulin. Corticosterone (A,B) and testosterone (C,D) plasma levels were measured at −30, −20, −10, 20, 40, 60, 80, 100 and 120 minutes after the i.c.v. infusion of Kp (3 nmol h^‐1^, pink circles), RFRP‐3 (50 pmol h^‐1^, green circles) or vehicle (NaCl 0.9%, open circles). Data are the mean ± SEM of n = 4‐6 for Kp and n = 5‐6 for RFRP‐3 treated animals per experimental group

The i.c.v. infusion of Kp increased plasma LH and testosterone concentrations, showing significant Treatment and Interaction effects (Figure 5A,C and Table 4). Post‐hoc analysis revealed that mean plasma levels of LH had already increased at 20 minutes (ie, in the first sample after the start of the i.c.v. infusion), but a statistically significant difference was only reached at 80 minutes (Figure 5A). Plasma testosterone levels showed statistically significant differences compared to the vehicle group 60, 100 and 120 minutes after the start of the i.c.v. infusion (Figure 5C). The i.c.v. RFRP‐3 infusions did not show any statistically significant difference in plasma LH or testosterone levels (Figure 5B,D and Table 4). Both RF‐amides did not have any significant effects on either on plasma corticosterone or insulin levels (Table 4 and Figure 6).

**TABLE 4:** Effects of i.c.v. kisspeptin (3 nmol) and i.c.v. RFRP‐3 (50 pmol) infusions on plasma hormone levels

Comparison	Parameter	Treatment	Time	Interaction
Vehicle vs kisspeptin	ΔLuteinising hormone (ng mL^‐1^)	***F*_1,8_** = **5.562** ***P* = 0.046**	***F*_8,64_** = **5.538** ***P* < 0.0001**	***F*_8,64_** = **5.209** ***P* < 0.0001**
	ΔTestosterone (nmol L^‐1^)	***F*_1,8_** = **6.928** ***P* = 0.030**	***F*_8,64_** = **6.235** ***P* < 0.0001**	***F*_8,64_** = **5.578** ***P* < 0.0001**
	ΔCorticosterone (ng mL^‐1^)	*F*_1,8_ = 0.9052 *P* = 0.369	***F*_8,64_** = **3.845** ***P* = 0.001**	*F*_8,64_ = 0.5208 *P* = 0.836
	ΔInsulin (ng mL^‐1^)	*F*_1,8_ = 2.627 *P* = 0.144	***F*_8,64_** = **2.869** ***P* = 0.009**	*F*_8,64_ = 1.293 *P* = 0.263
	ΔGlucose (mmol L^‐1^)	*F*_1,8_ = 0.9598 *P* = 0.356	***F*_8,64_** = **3.048** ***P* = 0.006**	*F*_8,64_ = 0.7549 *P* = 0.643
	ΔEndogenous glucose production (µmol kg·min^‐1^)	*F*_1,8_ = 3.481 *P* = 0.099	*F*_8,64_ = 1.090 *P* = 0.382	***F*_8,64_** = **2.204** ***P* = 0.039**
Vehicle vs RFRP3	ΔLuteinising hormone (ng mL^‐1^)	*F* (1, 9) = 0.2578 *P* = 0.624	***F*_8,72_** = **4.828** ***P* < 0.0001**	*F*_8,72_ = 0.6371 *P* = 0.744
	ΔTestosterone (nmol L^‐1^)	*F* (1, 9) = 0.1266 *P* = 0.730	*F*_8,72_ = 0.06769 *P* = 0.999	*F*_8,72_ = 0.2284 *P* = 0.985
	ΔCorticosterone (ng mL^‐1^)	*F* (1, 9) = 2.667 *P* = 0.137	***F*_8,72_** = **3.698** ***P* = 0.001**	*F*_8,72_ = 0.9926 *P* = 0.449
	ΔInsulin (ng mL^‐1^)	*F* (1, 9) = 0.5046 *P* = 0.496	*F*_8,72_ = 1.163 *P* = 0.334	*F*_8,72_ = 0.9767 *P* = 0.461
	ΔGlucose (mmol L^‐1^)	*F* (1, 9) = 0.2453 *P* = 0.632	***F*_8,72_** = **4.245** ***P* = 0.0003**	*F*_8,72_ = 0.3165 *P* = 0.957
	ΔEndogenous glucose production (µmol kg·min^‐1^)	*F* (1, 9) = 2.746 *P* = 0.132	*F*_8,72_ = 1.890 *P* = 0.075	*F*_8,72_ = 2.069 *P* = 0.050

Significant values are indicated in bold.

#### 3.2.2 Glycaemia and EGP after RF‐amide i.c.v. infusion

**FIGURE 7:**
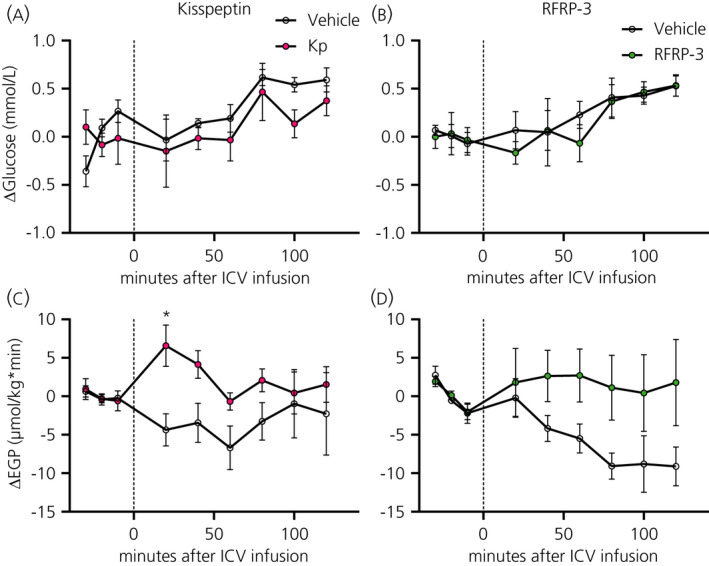
Effect of central kisspeptin (Kp) and RFRP‐3 on blood glucose and endogenous glucose production (EGP). Blood glucose (A,B) and EGP (C,D) were measured at −30, −20, −10, 20, 40, 60, 80, 100 and 120 minutes after the i.c.v. infusion of Kp (3 nmol h^‐1^, closed circles), RFRP3 (50 pmol h^‐1^, closed squares) or vehicle (NaCl 0.9%, open circles and open squares). Data are the mean ± SEM of n = 10 for Kp and n = 11 for RFRP‐3 treated animals per experimental group. *P < 0.05 statistical difference between Kp and vehicle after two‐way RM ANOVA and Bonferroni's post‐hoc honestly significance test

Both RF‐amides did not result in any significant changes in blood glucose levels, but the i.c.v. administration of Kp resulted in a significant increase of EGP as attested by a significant Interaction effect (*P* = 0.0385). Post‐hoc analysis showed that this increase started 20 minutes after the start of the i.c.v. infusion (Figure 7C and Table 4). The i.c.v. administration of RFRP‐3 also tended to increase EGP as indicated by the borderline significant Interaction effect (*P* = 0.050) (Figure 7D and Table 4).

### 3.3 Central targets of i.c.v. Kp and RFRP‐3

At the end of the Experiment 1, rats were given a third i.c.v. injection with either vehicle or one of the RF‐amides and, 1 hour later, animals were perfused and perfusion fixed brains processed for immunostaining.

#### 3.3.1 Activation of the median preoptic nucleus (MnPO) in response to Kp

**FIGURE 8:**
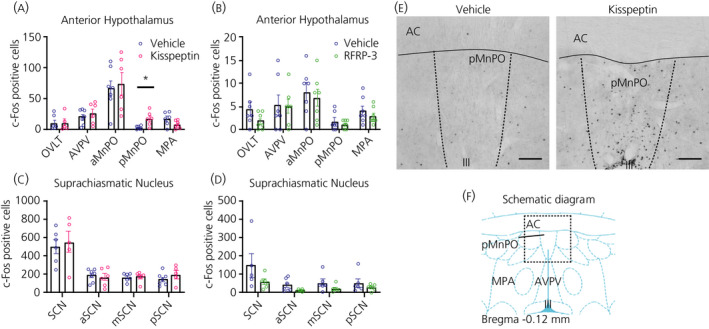
Effect of i.c.v. kisspeptin (Kp) and RFRP‐3 on c‐Fos expression in the male Wistar rat hypothalamus. The number of c‐Fos expressing cells was evaluated in various brain areas (ie, OVLT, AVPV, aMnPO, pMnPO and MPA) of the anterior hypothalamus (A, B) and anterior, medial and posterior parts of the suprachiasmatic nuclei (C, D), 60 min after the i.c.v. injection of Kp (3 nmol), RFRP‐3 (250 pmol) or vehicle (NaCl 0.9%). (E) Showing representative images of c‐Fos staining in the median preoptic nucleus (pMnPO) of animals injected with vehicle (left) or Kp (right). (F) Showing a schematic diagram obtained from the rat brain atlas (Paxinos and Watson) of the preoptic nuclei at a level equivalent to bregma −0.12 mm.^21^ Data represent the mean ± SEM of n = 5 ‐ 7 animals, with scattered dots representing individual values per animal. *P < 0.05 statistical difference between Kp and vehicle after Student's *t*‐test. III, third ventricle; AC, anterior comissura; aMnPO, anterior part of the median preoptic nucleus; AVPV, anteroventral periventricular nucleus; MPA, medial preoptic area; OVLT, organum vasculosum of the laminae terminalis; pMnPO, posterior part of the median preoptic nucleus; SCN, suprachiasmatic nucleus. Scale bar =200 µm

From all the brain regions analysed for c‐Fos immunoreactivity (Figure 8), only the posterior part of the median preoptic area showed a significant increase in the number of c‐Fos positive cells after the i.c.v. Kp treatment (*P* = 0.014) (Figure 8A). RFRP‐3 injections did not result in any significant increase in c‐Fos immunostaining in the investigated brain areas; indeed, in most brain areas, the amount of c‐Fos tended to decrease (Figure 8B,D), including in the suprachiasmatic nucleus in line with previously published results.^30^

### 3.4 POMC neurones respond to Kp

**FIGURE 9:**
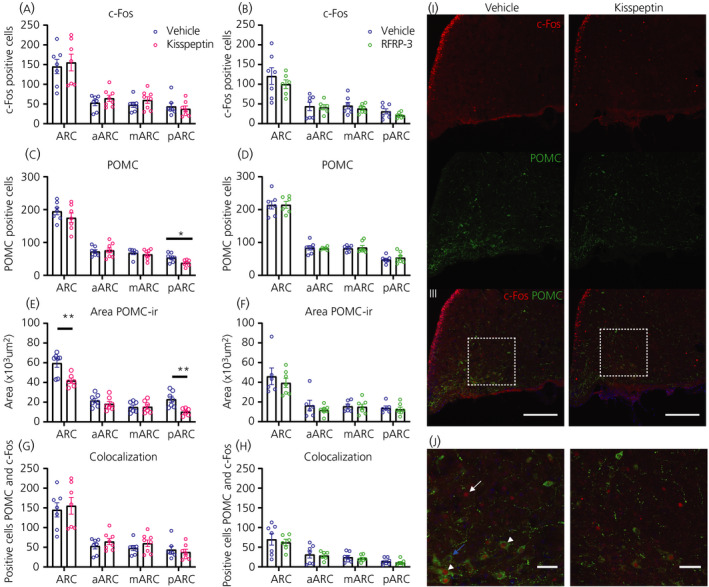
Effect of i.c.v. kisspeptin (Kp) and RFRP‐3 on neuronal activity and POMC immunoreactivity in the arcuate nucleus. Pro‐opiomelanocortin (POMC) neuronal activity was evaluated 1 hour after i.c.v. administration of Kp (3 nmol), RFRP‐3 (250 pmol) or vehicle (NaCl 0.9%), as the number of c‐Fos positive cells (A,B), number of POMC‐immunoreactive cells (C,D), area of coverage of POMC immunoreactivity (E,F) and number of POMC‐immunoreactive neurones expressing c‐Fos (G,H). Representative images (I) of double immunofluorescence for c‐Fos (red) and POMC (green) in the posterior arcuate nucleus(scale bar = 100 µm). (J) Zoom image showing positive c‐Fos (white arrow), POMC neurones (blue arrows) or co‐localisation (white arrowhead; Scale bar = 20 µm). Left panels: vehicle‐treated animals; right panels: Kp‐injected animals. 4ʹ,6‐Diamidino‐2‐phenylindole (blue) was used to stain nuclei. Data in (A) to (H) represent the mean ± SEM of n = 7 to 8 animals per experimental group and the scattered dots represent individual data values. **P < 0.01, *P < 0.05 statistical difference between Kp and vehicle after Student's *t*‐test. ARC, arcuate nucleus; aARC, anterior ARC; mARC, medial ARC; pARC, posterior ARC

POMC expression was analysed in the ARC of Kp‐ and RFRP‐3‐treated rats (Figure 9) because most of the POMC neurones have been reported to express RF‐amide receptors.^17^ Kp induced a significant decrease in the number of POMC‐immunoreactive cells in the posterior part of ARC (*P* = 0.024) (Figure 9C) . Also, total POMC immunoreactivity in the ARC was reduced, which was mainly a result of a decrease in its posterior part (*P* = 0.004) (Figure 9E). The number of POMC cells expressing c‐Fos was not modified (Figure 9G). RFRP‐3 injections had no significant effect on ARC c‐Fos or POMC expression (Figure 9B,D,F,H).

## 4 DISCUSSION

The present study reports a clear anorexigenic effect of central Kp in ad lib. fed rats, which was associated with a significant decrease in RER. By contrast to Kp, RFRP‐3 did not display any significant orexigenic effects. Regarding glucose metabolism, both RF‐amides had a stimulatory effect on endogenous glucose production, although this effect was only significant for Kp. In agreement with these data, central administration of Kp, but not RFRP‐3, changed neuronal activity in the preoptic nucleus of the hypothalamus. Together, these results provide further evidence that Kp is not only a very potent hypothalamic activator of reproduction, but also part of the hypothalamic circuit controlling glucose and energy metabolism, which makes the Kp system ideally suited for ensuring an optimal match of energy metabolism with the metabolic needs of reproduction. The metabolic effects of RFRP‐3 on the other hand appear to be very much species dependent, just like its reproductive effects.

The hormone measurements showed that i.c.v. administration of both Kp and RFRP‐3 was effective, with each reaching their brain targets. The increased activity of the HPG axis upon i.c.v. Kp, as shown by the marked increase in plasma testosterone concentrations, is in line with that reported before in rats,^31^ as well as after i.v. administration in humans.^32^ Intracerebrovascular administration of RFRP‐3 also increased circulating testosterone levels, but to a lesser extent. The different effect of RFRP‐3 in Experiments 1 and 2 is most likely a result of the different doses used (ie, 250 pmol in Experiment 1 and 50 pmol in Experiment 2). Although RFRP‐3 was initially discovered in birds and named gonadotrophin‐inhibitory hormone for its capacity to inhibit LH levels,^33^ recent studies have reported an activation of the HPG axis in male mice and hamsters.^34^, ^35^ Therefore, our data confirm that RFRP‐3 can induce activation of the HPG axis in male rodents.

Although the currently reported anorexic effect of central Kp in ad libitum fed rats confirms earlier observations in mice, rats and Jerboas,^10^, ^12^, ^36^ in the very first studies, no significant effects on feeding behaviour were found,^31^, ^37^ probably because the anorexic effect of Kp is only minor. In the present study, the anorexigenic effect of Kp was associated with a decrease in RER. This may be explained by the fact that longer periods of fasting induce an increase in lipid oxidation, which results in lower RER values. However, when comparing Figures 3A and 4B, it appears that the RER starts going down already much earlier (ie, immediately after the i.c.v. injection of Kp). On the other hand, a closer look at Figure 3C reveals that also the anorexigenic effect is apparent immediately after the i.c.v. injection of Kp. This indicates that Kp simultaneously decreases food intake and stimulates lipid oxidation, causing a decrease of RER. The effect of Kp appears to be rather specific for food intake because we found no changes in energy expenditure and locomotor activity, which is in line with the previous report of Thompson et al^31^. Kiss1R KO mice showed not only a higher body weight, but also reduced energy expenditure, lower locomotor activity and reduced thermogenesis.^5^, ^6^, ^38^, ^39^ However, this difference may be a result of the different experimental settings (ie, acute i.c.v. vs chronic and developmental KO effects). Notably, selective KO of the Kiss1R from brown adipose tissue caused a reduction in body weight and increased energy expenditure,^38^ clearly illustrating the tissue‐specific functions of Kp.

To elucidate the neuronal targets that might be involved in the anorexigenic effect of Kp, we investigated the activity of POMC neurones because they are the main neuronal population in the ARC expressing the Kiss1R^17^ and are very well characterised for their inhibitory role on food intake and body weight.^40^ We found that Kp significantly decreased POMC immunoreactivity, demonstrating that i.c.v. Kp was indeed affecting the activity of the POMC neurones. The decreased POMC immunoreactivity indicates that Kp increased the cleavage, transport and release of processed neuropeptides (ie alpha‐melanocortin stimulating hormone [α‐MSH]). This hypothesis is supported by studies in mice demonstrating a direct activation of POMC neurones by Kp^41^, ^42^ and by our own studies in hamsters showing that Kp increases POMC mRNA levels.^7^ In line with the above physiological data, central administration of α‐MSH has been shown to simultaneously decrease food intake and increase lipid oxidation.^43^ In addition, reduction of brain‐melanocortin signalling consistently results in an increased RER^44^, ^45^, ^46^, ^47^ indicative of reduced lipid utilisation. Unfortunately, we found no increased c‐Fos expression or POMC/c‐Fos co‐localisation in the ARC, which would have provided further support for the Kp‐induced activation of POMC neurones. The stimulatory effect of Kp on POMC neuronal activity has been clearly demonstrated with electrophysiological techniques, whrereas Kp effects on NPY neuronal activity are more equivocal.^41^, ^42^ Surprisingly, as far as we are aware, increased Kp‐induced expression of c‐Fos has only been demonstrated for NPY neurones.^48^

POMC and NPY neurones in the ARC exhibit antagonistic effects on food intake.^49^, ^50^ Indeed, besides the above mentioned stimulatory effect of Kp on POMC neurones, Kp has been reported to inhibit NPY neuronal activity.^41^, ^42^ However, opposite effects were observed in ovariectomised sheep (ie, increased NPY expression and reduced POMC expression),^51^ whereas, in female Jerboas, the anorexigenic effect of Kp was accompanied by an increased expression of POMC, but no changes in NPY expression.^10^ Therefore, in future experiments, it will be interesting to analyse whether central Kp also changes NPY expression. Notably, the central injection of Kp increased c‐Fos expression in the MnPO, a region known to receive a direct POMC innervation from the ARC.^52^ Pharmacological manipulations of α‐MSH signalling to the MnPO changed both c‐Fos expression and core body temperature. Recently, it was shown that ARC Kp neurones are also part of the neural circuit modulating the circadian control of body temperature.^53^ In addition, Kp is also known to change the expression of other (an)orexigenic peptides, such as brain‐derived neurotrophic factor, melanin‐concentrating hormone , nesfatin‐1 and oxytocin.^36^, ^54^, ^55^ Therefore, at present, no final conclusions can be made on the appetite‐regulating pathways of Kp.

The currently observed effects of Kp on glucose metabolism appear to be in line with the glucose intolerance observed in Kiss1r KO mice.^5^, ^38^ In the present study, Kp increased glucose production, but plasma glucose levels were not changed indicating a concomitant increase in glucose tolerance. On the other hand, the increased glucose production does not appear to be in line with the decreased RER observed upon the central administration of Kp (ie, increased lipid oxidation). However, the experimental set‐ups were different (i.c.v. bolus injection plus ad libitum feeding in Experment 1 and i.c.v. continuous infusion plus fasting in Experiment 2) and the extra glucose produced does not necessarily have to be oxidised, but can also be stored in muscle or adipose tissue. Clearly, much still remains unknown regarding the central and peripheral glucoregulatory effects of Kp.

Surprisingly, RFRP‐3 did not display any significant orexigenic effects. Although RFRP‐3 did increase testosterone levels at the highest dose tested, no significant effects on food intake or energy metabolism were found. These findings are in clear contrast to that reported previously in other mammalian species,^7^, ^9^, ^10^, ^11^ as well as in ad lib. fed Wistar rats.^8^, ^9^ However, except for the different species used, also the variance in doses, RFRP‐3 preparation and experimental set‐up used (eg, i.c.v. bolus injections vs 2‐hour or 5‐day infusions or fed vs fasted status) might be responsible for these apparent discrepancies. Regarding glucose metabolism, little is known about possible effects of RFRP‐3. Intraperitoneal administration of RFRP‐3 has been shown to change circulating glucose concentrations, as well as insulin receptor and glucose transporter expression in testis and adipose tissue,^13^, ^56^ and a lack of the RFRP‐3 receptor in NPFF1R KO mice worsened the metabolic impact of a high‐fat diet on glucose homeostasis in male but not female mice.^6^ RFRP‐3 neurones also project to the NPY‐ and POMC‐containing neurones in the ARC^57^; thus, in view of the reported opposite effects on POMC neuronal activity,^42^ the comparable effects on glucose metabolism may appear unexpected. Unfortunately, as a result of the lack of clear c‐Fos activation, it remains unclear which of these neuronal populations could be the target for the metabolic effects of RFRP‐3, although the absence of c‐Fos activation could be related to the often reported inhibitory effect of RFRP‐3.^30^, ^42^, ^58^, ^59^

In conclusion, in male Wistar rats, central administration of Kp caused a clear activation of the HPG axis, reduced food intake, increased lipid oxidation and increased glucose production. The changes observed in ARC POMC immunoreactivity and MnPO c‐Fos expression, support the idea that these metabolic changes are caused by a Kp‐induced activation of POMC neurones. On the other hand, central administration of RFRP‐3 caused a mild activation of the HPG axis, but did not result in any significant metabolic effects.

## CONFLICT OF INTERESTS

The authors declare that they have no conflicts of interest.

## AUTHOR CONTRIBUTIONS

**Fernando Cazarez Marquez:** Conceptualisation; Investigation; Methodology; Writing – original draft. **Jitske Eliveld:** Investigation; Methodology. **Wayne Ritsema:** Investigation; Methodology. **Ewout Foppen:** Investigation; Methodology; Project administration. **Yvonne Bossenbroek:** Methodology. **Simone Pelizzari:** Investigation. **Valérie Simonneaux:** Funding acquisition; Supervision; Writing – review & editing. **A Kalsbeek:** Conceptualisation; Funding acquisition; Project administration; Supervision; Writing – review & editing.

### PEER REVIEW

The peer review history for this article is available at https://publons.com/publon/10.1111/jne.12973.

## Supporting information

Figure S1

Figure S2

Figure S3

Figure S4

Figure S5

Table S1‐S3

## Data Availability

The data that support the findings of this study are available from the corresponding author upon reasonable request.
