# Supplementary material for: Role of central kisspeptin and RFRP‐3 in energy metabolism in the male Wistar rat
Source: J Neuroendocrinol. 2021 May 7;33(7):e12973. doi: 10.1111/jne.12973 (PMC8365661; doi:10.1111/jne.12973)
Supplement: Supplementary file 6 — Table S1‐S3 [file JNE-33-e12973-s004.docx]

| **Table S1. Effects of ICV RFRP3 injection (50 pmol) on metabolic outcomes** | | | | |
| --- | --- | --- | --- | --- |
| Comparison | Parameter | *Treatment* | *Time* | *Interaction* |
| Baseline vs Vehicle icv | Cumulative food intake | F (1, 4) = 1.509  P=0.287 | **F (95, 380) = 318.6**  **P<0.0001** | **F (95, 380) = 1.342**  **P=0.029** |
|  | Cumulative water  intake | **F (1, 4) = 47.06**  **P=0.002** | **F (95, 380) = 162.0**  **P<0.0001** | **F (95, 380) = 2.262**  **P<0.0001** |
|  | Locomotor activity | F (1, 4) = 1.831  P=0.248 | **F (94, 376) = 3.882**  **P<0.0001** | F (94, 376) = 1.248  P=0.078 |
|  | RER | F (1, 4) = 0.3141  P=0.605 | **F (94, 376) = 11.56**  **P<0.0001** | F (94, 376) = 1.037  P=0.398 |
|  | Heat | F (1, 4) = 0.1408  P=0.727 | **F (94, 376) = 3.893**  **P<0.0001** | F (94, 376) = 1.257  P=0.071 |
| Baseline vs  RFRP3 50 pmol icv | Cumulative food intake | F (1, 4) = 1.274  P=0.322 | **F (95, 380) = 99.65**  **P<0.0001** | **F (95, 380) = 1.705**  **P=0.0002** |
|  | Cumulative water  intake | F (1, 4) = 0.04041  P=0.851 | **F (95, 380) = 93.83**  **P<0.0001** | **F (95, 380) = 2.338**  **P<0.0001** |
|  | Locomotor activity | F (1, 4) = 0.1634  P=0.707 | **F (94, 376) = 2.264**  **P<0.0001** | **F (94, 376) = 1.474**  **P=0.006** |
|  | RER | F (1, 4) = 1.436  P=0.297 | **F (94, 376) = 6.087**  **P<0.0001** | F (94, 376) = 0.5616  P=0.999 |
|  | Heat | F (1, 4) = 1.219  P=0.332 | **F (94, 376) = 3.131**  **P<0.0001** | F (94, 376) = 0.9944  P=0.501 |
| Vehicle icv vs  RFRP3 50 pmol icv | Cumulative food intake | F (1, 4) = 1.810  P=0.250 | **F (95, 380) = 137.9**  **P<0.0001** | **F (95, 380) = 1.781**  **P<0.0001** |
|  | Cumulative water  intake | F (1, 4) = 0.06417  P=0.813 | **F (95, 380) = 176.8**  **P<0.0001** | F (95, 380) = 0.4051  P>0.999 |
|  | Locomotor activity | F (1, 4) = 0.1245  P=0.742 | **F (94, 376) = 3.323**  **P<0.0001** | F (94, 376) = 1.093  P=0.280 |
|  | RER | F (1, 4) = 1.049  P=0.364 | **F (94, 376) = 9.408**  **P<0.0001** | F (94, 376) = 1.360  P=0.024 |
|  | Heat | F (1, 4) = 0.001084  P=0.975 | **F (94, 376) = 4.278**  **P<0.0001** | F (94, 376) = 0.9422  P=0.629 |

| **Table S2. Effects of ICV RFRP3 injection (250 pmol) on metabolic outcomes** | | | | |
| --- | --- | --- | --- | --- |
| Comparison | Parameter | *Treatment* | *Time* | *Interaction* |
| Baseline vs Vehicle icv | Cumulative food intake | F (1, 3) = 0.8773  P=0.418 | **F (95, 285) = 73.28**  **P<0.0001** | **F (95, 285) = 2.251**  **P<0.0001** |
|  | Cumulative water  intake | F (1, 3) = 4.311  P=0.130 | **F (95, 285) = 135.2**  **P<0.0001** | **F (95, 285) = 2.009**  **P<0.0001** |
|  | Locomotor activity | **F (1, 3) = 16.25**  **P=0.027** | **F (94, 282) = 3.786**  **P<0.0001** | **F (94, 282) = 1.431**  **P=0.014** |
|  | RER | F (1, 3) = 3.866  P=0.144 | **F (94, 282) = 5.222**  **P<0.0001** | F (94, 282) = 0.6330  P=0.995 |
|  | Heat | **F (1, 3) = 17.02**  **P=0.026** | **F (94, 282) = 3.003**  **P<0.0001** | F (94, 282) = 0.7960  P=0.903 |
| Baseline vs  RFRP3 250 pmol icv | Cumulative food intake | F (1, 3) = 2.953  P=0.184 | **F (95, 285) = 153.9**  **P<0.0001** | F (95, 285) = 1.303  P=0.051 |
|  | Cumulative water  intake | F (1, 3) = 0.9880  P=0.394 | **F (95, 285) = 242.7**  **P<0.0001** | F (95, 285) = 0.4465  P=0.792 |
|  | Locomotor activity | F (1, 3) = 4.331  P=0.129 | **F (94, 282) = 4.507**  **P<0.0001** | **F (94, 282) = 1.431**  **P=0.013** |
|  | RER | F (1, 3) = 3.944  P=0.141 | **F (94, 282) = 7.620**  **P<0.0001** | F (94, 282) = 0.9631  P=0.577 |
|  | Heat | F (1, 3) = 0.8053  P=0.436 | **F (94, 282) = 4.301**  **P<0.0001** | F (94, 282) = 1.076  P=0.320 |
| Vehicle icv vs  RFRP3 250 pmol icv | Cumulative food intake | F (1, 3) = 0.2429  P=0.656 | **F (95, 285) = 69.67 P<0.0001** | F (95, 285) = 0.7546 P=0.946 |
|  | Cumulative water  intake | F (1, 3) = 0.1799  P=0.700 | **F (95, 285) = 113.1 P<0.0001** | F (95, 285) = 1.232  P=0.098 |
|  | Locomotor activity | F (1, 3) = 0.9357  P=0.405 | **F (94, 282) = 5.232 P<0.0001** | F (94, 282) = 0.7562 P=0.944 |
|  | RER | F (1, 3) = 0.3088  P=0.617 | **F (94, 282) = 4.248 P<0.0001** | **F (94, 282) = 1.636**  **P=0.001** |
|  | Heat | F (1, 3) = 0.0003453  P=0.986 | **F (94, 282) = 4.726 P<0.0001** | F (94, 282) = 0.8825 P=0.756 |

| **Table S3. Effects of ICV RFRP3 injection (50and 250 pmol combined) on metabolic outcomes** | | | | |
| --- | --- | --- | --- | --- |
| Comparison | Parameter | *Treatment* | *Time* | *Interaction* |
| Baseline vs Vehicle icv | Cumulative food intake | F (1, 8) = 2.187  P=0.178 | **F (95, 760) = 300.8**  **P<0.0001** | **F (95, 760) = 1.681**  **P=0.0001** |
|  | Cumulative water  intake | **F (1, 8) = 26.84**  **P=0.0008** | **F (95, 760) = 294.6**  **P<0.0001** | **F (95, 760) = 3.745**  **P<0.0001** |
|  | Locomotor activity | **F (1, 8) = 8.525**  **P=0.019** | **F (94, 752) = 5.711**  **P<0.0001** | F (94, 752) = 1.005  P=0.471 |
|  | RER | F (1, 8) = 1.187  P=0.308 | **F (94, 752) = 13.53**  **P<0.0001** | F (94, 752) = 0.9255  P=0.675 |
|  | Heat | F (1, 8) = 1.962  P=0.199 | **F (94, 752) = 5.702**  **P<0.0001** | F (94, 752) = 1.035  P=0.396 |
| Baseline vs  RFRP3 icv | Cumulative food intake | F (1, 8) = 2.949  P=0.124 | **F (95, 760) = 248.7**  **P<0.0001** | **F (95, 760) = 2.499**  **P<0.0001** |
|  | Cumulative water  intake | F (1, 8) = 0.8974  P=0.371 | **F (95, 760) = 262.1**  **P<0.0001** | **F (95, 760) = 1.346**  **P=0.021** |
|  | Locomotor activity | F (1, 8) = 1.011  P=0.344 | **F (94, 752) = 5.306**  **P<0.0001** | **F (94, 752) = 1.623**  **P=0.0004** |
|  | RER | F (1, 8) = 4.012  P=0.080 | **F (94, 752) = 13.66**  **P<0.0001** | F (94, 752) = 0.5677  P=0.999 |
|  | Heat | F (1, 8) = 1.759  P=0.221 | **F (94, 752) = 6.548**  **P<0.0001** | F (94, 752) = 1.178  P=0.1310 |
| Vehicle icv vs  RFRP3 icv | Cumulative food intake | F (1, 8) = 0.6190  P=0.454 | **F (95, 760) = 217.7**  **P<0.0001** | F (95, 760) = 0.8117  P=0.899 |
|  | Cumulative water  intake | F (1, 8) = 0.1142  P=0.744 | **F (95, 760) = 294.5**  **P<0.0001** | F (95, 760) = 0.2855  P>0.999 |
|  | Locomotor activity | F (1, 8) = 0.004303  P=0.949 | **F (94, 752) = 6.274**  **P<0.0001** | F (94, 752) = 0.5768  P=0.999 |
|  | RER | F (1, 8) = 0.1417  P=0.716 | **F (94, 752) = 12.41**  **P<0.0001** | F (94, 752) = 0.7319  P=0.971 |
|  | Heat | F (1, 8) = 0.001425  P=0.971 | **F (94, 752) = 7.271**  **P<0.0001** | F (94, 752) = 0.8236  P=0.881 |
